# Skirting the pitfalls: a clear-cut nomenclature for H3K4 methyltransferases

**DOI:** 10.1111/cge.12050

**Published:** 2012-11-27

**Authors:** N Bögershausen, E Bruford, B Wollnik

**Affiliations:** a Institute of Human GeneticsCologne, Germany; b Center for Molecular Medicine Cologne (CMMC)Cologne, Germany; c Cologne Excellence Cluster on Cellular Stress Responses in Aging-Associated Diseases (CECAD), University of CologneCologne, Germany; d HUGO Gene Nomenclature Committee (HGNC), European Bioinformatics Institute (EMBL-EBI), Wellcome Trust Genome CampusHinxton, Cambridgeshire, UK

**Keywords:** chromatin-modifying enzymes, Kabuki syndrome, *KMT2*, *KMT2B*, *KMT2D*, *MLL*, *MLL2*, *MLL4*, nomenclature

## Abstract

To unravel the system of epigenetic control of transcriptional regulation is a fascinating and important scientific pursuit. Surprisingly, recent successes in gene identification using high-throughput sequencing strategies showed that, despite their ubiquitous role in transcriptional control, dysfunction of chromatin-modifying enzymes can cause very specific human developmental phenotypes. An intriguing example is the identification of *de novo* dominant mutations in *MLL2* as a cause of Kabuki syndrome, a well-known congenital syndrome that is associated with a very recognizable facial gestalt. However, the existing confusion in the nomenclature of the human and mouse *MLL* gene family impedes correct interpretation of scientific findings for these genes and their encoded proteins. This Review aims to point out this nomenclature pitfall, to explain its historical background, and to promote an unequivocal nomenclature system for chromatin-modifying enzymes as proposed by Allis et al. (2007).

**Conflict of interest:**

The authors declare no conflict of interest

## The *MLL* nomenclature: more trouble than it's worth?

*MLL2* (MIM 602113; NM_003482, chromosome 12q13.12), a gene encoding a histone 3 lysine 4 (H3K4) methyltransferase of the trithorax group, has recently become of high interest for clinical and molecular genetics since the identification of *de novo* dominant *MLL2* mutations as the major cause of Kabuki syndrome 1. Previously, a significant amount of research had already been dedicated to enzymes of chromatin-modifying function involved in various processes of transcriptional regulation in development and physiological states. However, the correct correlation and interpretation of these scientific findings are hindered by a major confusion in gene nomenclature that needs to be resolved.

In 1998 the HUGO Gene Nomenclature Committee (HGNC) approved the name *MLL2* for the human gene located on chromosome 12q13.12. However, FitzGerald and Diaz described another member of the human *MLL* gene family that was located on chromosome 19q13.12 in 1999 and also named this gene *MLL2* 2. Since then both genes have been referred to as *MLL2* somewhat inconsistently throughout the literature and, to enhance confusion, both genes have also been referred to as *MLL4*. In particular the mouse orthologue of the human chromosome 12 gene (MGI: 2682319, chromosome 15), which is approved as *Mll2* by the Mouse Genomic Nomenclature Committee (MGNC), has been frequently referred to as *Mll4* in the literature, for example in a paper by Cho et al. describing a protein complex with H3K4 methyltransferase activity (ASCOM) that actually contains *Mll2*, *Mll3* and several other binding partners 3.

The designation *MLL4* is currently ascribed to the human chromosome 19 gene at the UCSC genome browser (http://genome.ucsc.edu; accession number NM_014727), but this name is not approved by the HGNC. Currently, there is no gene in human or mouse approved as *MLL4*. The mouse orthologue of the human chromosome 19 gene is assigned as *Wbp7* (MGI: 109565, chromosome 7) and its gene product sometimes referred to as MLL4 (see UCSC genome browser). But controversially it is also referred to as MLL2 in the literature, for example in a publication by Glaser et al. on the role of ‘MLL2’ in embryonic development 4, or a publication on gene expression in breast and colon cancer by Natarajan et al. 5. Consequently, these data on the chromosome 19 gene could be incorrectly interpreted as attributable to the human chromosome 12 gene if one is not aware of the confusing nomenclature.

This mix-up of nomenclature might become evident on close inspection of a paper if accession numbers are provided, but how can anyone interpret findings in publications that do not give any accession numbers? How can anyone determine whether the results deal with the gene/protein one is interested in? The group of Issaeva et al. tried to overcome this confusion by using synonyms, for example, *ALR* for *MLL2* 6, but since *ALR* has also been used as a synonym for both genes 7, the *ALR* nomenclature is just as incoherent and confusing.

## A resolute solution: the KMT nomenclature

There is an urgent need for an intelligible nomenclature of genes coding H3K4 methyltransferases. Instead of swapping gene symbols in an already confusing situation, we propose in agreement with the HGNC and the MGNC (Dr Elspeth Bruford, personal communication), to completely replace the *MLL* nomenclature system. Although it is widely used by researchers and clinicians, especially for the *MLL*/*Mll1* gene, the *MLL* nomenclature is outdated and does not give credit to the complex function of the MLL group proteins; instead it suggests a link with myeloid leukaemia that is not valid for most members of the family. As for *MLL2,* new sequencing technologies have recently provided evidence for the implication of *MLL* and *MLL3* in the pathogenesis of human developmental syndromes 8, 9, emphasizing their importance for the field of human genetics, and other family members are bound to follow. Establishment of an unequivocal nomenclature system, based on protein function, would help to avoid misunderstandings in the future.

**Fig. 1:**
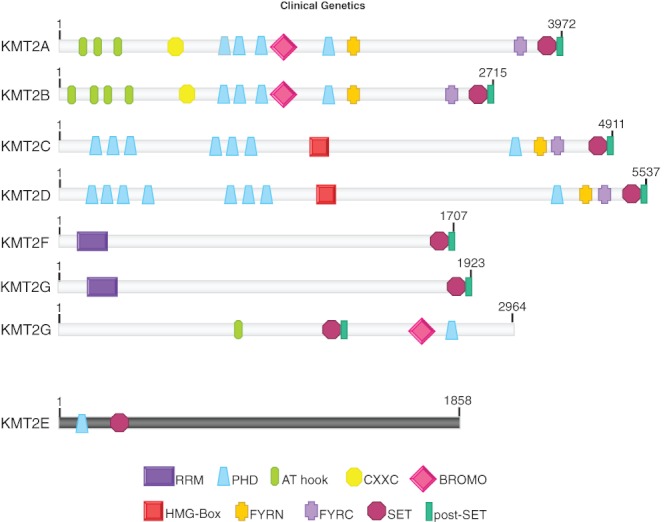
Schematic representation of KMT2 group proteins. Proteins are shown as grey bars, domains are indicated by coloured symbols (colour code in the figure), digits at the beginning and end of the bars indicate the number of amino acids. KMT2E is represented separately as a dark grey bar with the same colour code for domains, to make clear that this protein is functionally different from the other family members but shows homology in domain structure. Information on domain architecture was according to Smith et al. 13 and the Human Protein Reference Database (http://www.hprd.org/index_html).

Previously, Allis et al. suggested a well-structured and systematic nomenclature for chromatin-modifying enzymes 10, including the MLL group of enzymes, and this nomenclature has since been endorsed by both HGNC and MGNC. This new nomenclature names and categorizes enzymes depending on their homology in sequence and domain structure, and groups them according to their chromatin-modifying function. Thus, lysine demethylases are termed K-demethylases (KDMs), lysine acyltransferases are named K-acyltransferases (KATs), and the lysine methyltransferases are classified as K-methyltransferases (KMTs). Consequently, the H3K4 methyltransferases are subsumed into the KMT2 group (Table 1; Fig. 1) and the group members are numbered by the order of published records. Naming these proteins as KMTs is correct for all the members except MLL5, which does not have an intrinsic methyltransferase activity 11. Nevertheless, given its homology to the other family members, we propose to reassign it as *KMT2E* in line with Allis et al. The suggested nomenclature has already found wide acceptance for other groups of chromatin-modifying enzymes, showing that changes in nomenclature do become acceptable with time. One prominent example is *KDM6A*, a lysine demethylase shown to cause Kabuki syndrome in case of intragenic or whole-gene deletions 12.

**Table 1:** Proposed nomenclature

	Old name					Function	Accession number	Chromosome
				
New name	Human	Mouse	*Drosophila melanogaster*	*Saccharomyces cervisiae*	*Schizosaccharomyces pombe*	All species	Human	Human
*KMT2*				*Set1*	*Set1*	H3K4-MT		
*KMT2A*	*MLL*	*Mll1*	*Trx*	—	—	H3K4-MT	NM_001197104	11q23.3
*KMT2B*	*MLL4*^a^	*Wbp7*	*Trx*	—	—	H3K4-MT	NM_014727	19q13.12
*KMT2C*	*MLL3*	*Mll3*	*Trr*	—	—	H3K4-MT	NM_170606	7q36.1
*KMT2D*	*MLL2*	*Mll2*	*Trr*	—	—	H3K4-MT	NM_003482	12q13.12
*KMT2E*^b^	*MLL5*^b^	*Mll5*	—	—	—	No MT-activity	NM_018682	7q22.3
*KMT2F*	*SETD1A*	*Setd1a*	—	—	—	H3K4-MT	NM_014712	16p11.2
*KMT2G*	*SETD1B*	*Setd1b*	—	—	—	H3K4-MT	NM_015048	12q24.21
*KMT2H*	*ASH1L*	*Ash1l*	*Ash1*	—	—	H3K4-MT	NM_018489	1q22

H3K4, histone 3 lysine 4; KMT, K-methyltransferases

a *MLL4* has been used for this gene but was never approved by HUGO Gene Nomenclature Committee.

b Although the encoded protein has no KMT activity, it is included in the classification based on its partial homology to other family members.

## Conclusion

The ambiguous *MLL* nomenclature is outdated and has been causing confusion for many years. The *KMT* nomenclature introduced by Allis et al. represents a comprehensible system, largely based on functional data and homology that is already commonly used for gene families other than the *MLL* group, such as the KDMs. Moreover, its link to the *MLL* nomenclature will always be easily trackable in the public domain, and thus the risk of creating further perturbation through establishment of this new nomenclature is negligible. We propose acceptance of the *KMT* nomenclature for chromatin-modifying enzymes and for the respective coding genes by the scientific community, especially for the *KMT2* gene family, in order to avoid misapprehension of scientific results in the future.

## References

[1] Ng SB, Bigham AW, Buckingham KJ (2010). Exome sequencing identifies *MLL2* mutations as a cause of Kabuki syndrome. Nat Genet.

[2] FitzGerald KT, Diaz MO (1999). MLL2: a new mammalian member of the trx/MLL family of genes. Genomics.

[3] Cho YW, Hong T, Hong S (2007). PTIP associates with MLL3- and MLL4-containing histone H3 lysine 4 methyltransferase complex. J Biol Chem.

[4] Glaser S, Lubitz S, Loveland KL (2009). The histone 3 lysine 4 methyltransferase, Mll2, is only required briefly in development and spermatogenesis. Epigenetics Chromatin.

[5] Natarajan TG, Kallakury BV, Sheehan CE (2010). Epigenetic regulator MLL2 shows altered expression in cancer cell lines and tumors from human breast and colon. Cancer Cell Int.

[6] Issaeva I, Zonis Y, Rozovskaia T (2007). Knockdown of *ALRMLL2*) reveals ALR target genes and leads to alterations in cell adhesion and growth. Mol Cell Biol.

[7] Lee S, Roeder RG, Lee JW (2009). Roles of histone H3-lysine 4 methyltransferase complexes in NR-mediated gene transcription. Prog Mol Biol Transl Sci.

[8] Jones WD, Dafou D, McEntagart M (2012). *De novo* Mutations in *MLL* Cause Wiedemann-Steiner syndrome. Am J Hum Genet.

[9] Kleefstra T, Kramer JM, Neveling K (2012). Disruption of an EHMT1-associated chromatin-modification module causes intellectual disability. Am J Hum Genet.

[10] Allis CD, Berger SL, Cote J (2007). New nomenclature for chromatin-modifying enzymes. Cell.

[11] Sebastian S, Sreenivas P, Sambasivan R (2009). MLL5, a trithorax homolog, indirectly regulates H3K4 methylation, represses cyclin A2 expression, and promotes myogenic differentiation. Proc Natl Acad Sci USA.

[12] Lederer D, Grisart B, Digilio MC (2012). Deletion of *KDM6A*, a histone demethylase interacting with MLL2, in three patients with Kabuki syndrome. Am J Hum Genet.

[13] Smith E, Lin C, Shilatifard A (2011). The super elongation complex (SEC) and MLL in development and disease. Genes Dev.

